# Feeding the fever: Complex host‐pathogen dynamics along continuous resource gradients

**DOI:** 10.1002/ece3.10315

**Published:** 2023-07-25

**Authors:** Elizabeth T. Borer, Amy E. Kendig, Robert D. Holt

**Affiliations:** ^1^ Department of Ecology, Evolution, and Behavior University of Minnesota Saint Paul Minnesota USA; ^2^ Agronomy Department University of Florida Gainesville Florida USA; ^3^ Minnesota Department of Natural Resources Minnesota Biological Survey Saint Paul Minnesota USA; ^4^ Department of Biology University of Florida Gainesville Florida USA

**Keywords:** bacteria, ecological theory, fungus, host‐pathogen, invertebrate, mathematical model, microbe, nitrogen, nutrient metals, phosphorus, plant, stoichiometry, vertebrate, virus

## Abstract

Food has long been known to perform dual functions of nutrition and medicine, but mounting evidence suggests that complex host‐pathogen dynamics can emerge along continuous resource gradients. Empirical examples of nonmonotonic responses of infection with increasing host resources (e.g., low prevalence at low and high resource supply but high prevalence at intermediate resources) have been documented across the tree of life, but these dynamics, when observed, often are interpreted as nonintuitive, idiosyncratic features of pathogen and host biology. Here, by developing generalized versions of existing models of resource dependence for within‐ and among‐host infection dynamics, we provide a synthetic view of nonmonotonic infection dynamics. We demonstrate that where resources jointly impact two (or more) processes (e.g., growth, defense, transmission, mortality, predation), nonmonotonic infection dynamics, including alternative states, can emerge across a continuous resource supply gradient. We review the few empirical examples that concurrently measured resource effects on multiple rates and pair this with a wide range of examples in which resource dependence of multiple rates could generate nonmonotonic infection outcomes under realistic conditions. This review and generalized framework highlight the likely generality of such resource effects in natural systems and point to opportunities ripe for future empirical and theoretical work.

## Graphical abstract

Nonmonotonic infection patterns have been observed along continuous resource gradients in a variety of ecosystems. By developing generalized models to focus on within‐ and among‐host infection processes, we demonstrate that nonmonotonic infection dynamics can arise when resources impact multiple processes simultaneously, resulting in switches in stability and alternative stable states along continuous resource supply gradients. Using these system‐independent models to motivate a survey of the empirical literature suggests that these countervailing processes and resulting dynamics are likely common in natural systems.

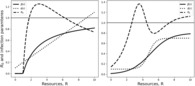

## 1 INTRODUCTION

Fasting is a great remedy of fever. John Withals, 1574

The relationship between food and health has a long history in human society, with an abundance of questionable products and dubious advice for improving health and a handful of time‐tested remedies. Nutrition also has been long acknowledged in mediating the symptoms of infectious disease for humans (Dunkin, 1937; Smith & McClung, 2021), livestock (Coop & Kyriazakis, 2001; Gilruth, 1932), and crops (Spencer, 1941). However, wild plant and animal populations commonly experience resource limitation, and livestock, crops, humans, and even wildlife are sometimes provided with excess resources, shaping the nutritional environment for both hosts and their pathogens. Importantly, because the host serves as the environment for its pathogens, host organisms and their pathogens share – and may compete for – a wide range of material resources that are required to support the metabolism, growth, defense, and reproduction of both infectious pathogens and their hosts (Box 1).

BOX 1 **Defining resources.**

For the purposes of the models and review, we follow Tilman (1982) by defining a resource as “any substance or factor, which can lead to increased [population] growth rates as its availability in the environment is increased, and which is consumed by an organism.” Tilman goes on to define “consumed” broadly as any use that reduces the availability of the resource for another organism (e.g., taking up space) (Tilman, 1982).

*Resource types*: Resources can be classified as biotic or abiotic. Biotic resources are organisms that reside, by definition, in a lower trophic level than their consumers. In contrast, abiotic resources do not consume other resources and include elemental nutrients, detritus, water, light, and space. Consumption of either biotic or abiotic resources potentially leads to intraspecific competition among consumers, inducing a consumer's carrying capacity. We constrain our current focus to resources affecting physiology or abundance of hosts, with a particular emphasis on resources that also can impact the physiology of their pathogens.

*Resource sources and uptake*: Resources may be either present in the environment or provided by humans. Humans may intentionally provision resources (e.g., bird feeders and fertilizers) or may unintentionally alter their concentration in the environment (e.g., nitrogen deposition, species introductions). While some sources provide a constant resource supply, others generate resource pulses. Hosts, our focal organisms, may take up resources in proportion to environmental supply or may select less common resources to meet nutritional needs. Finally, biotic resources may resist consumption through physical, chemical, or behavioral defenses.

*Mathematical representation*: We represent biotic resource growth using a logistic growth function (Box 4), which includes an intrinsic growth rate and carrying capacity. While this formulation accurately describes species with intraspecific competition (e.g., plants, animals, microbes), it is not the best description of abiotic resources (Boxes 2 and 3). For example, terrestrial and aquatic plants take up elemental nutrients (e.g., nitrogen, phosphorus, potassium, silica) and animals consume resources provided by humans (i.e., provisions). Abiotic resources are better described with a constant supply rate (e.g., *s*) or an input rate that depends on the concentration of the resource in the system relative to a maximum amount (e.g., *R*_max_−*R*). Often (albeit not always), comparable phenomena to those we highlight in this review arise for either process of resource renewal.

Given the importance of resources to host health and population dynamics, this topic has received extensive attention. Reviews of a vast number of empirical studies of both animal and plant hosts have demonstrated the impact of nutritional resources on various aspects of host health and pathogen dynamics across the tree of life (Becker et al., 2015; Huber & Watson, 1974; Humphries et al., 2021; Johnson et al., 2010; Pike et al., 2019; Smith et al., 2005; Smith & Holt, 1996). Examples illustrate responses in infectious disease ranging from strong reductions to substantial increases in pathogen prevalence or virulence. Models describing the impact of resources on pathogen populations or within‐host infection dynamics have pointed to key relationships that may govern such empirical observations. In particular, population‐scale models have demonstrated that elevated resource supply to hosts may induce nonintuitive impacts on the host's or pathogen's fitness (e.g., Béchette et al., 2013; Hurtado et al., 2014; Levin et al., 1977; Strauss et al., 2019). At the within‐host scale, for hosts and pathogens with overlapping resource requirements, altering the availability or environmental supply of resources to host organisms could potentially alter the metabolic state of the host and its responses to an invading pathogen (Pell et al., 2019; Smith & Holt, 1996; Weinbauer, 2004) – or may alter the balance between pathogen growth rate and the growth rate of key host cells (Smith et al., 2015), with emergent consequences at the host population level.

**FIGURE 1:**
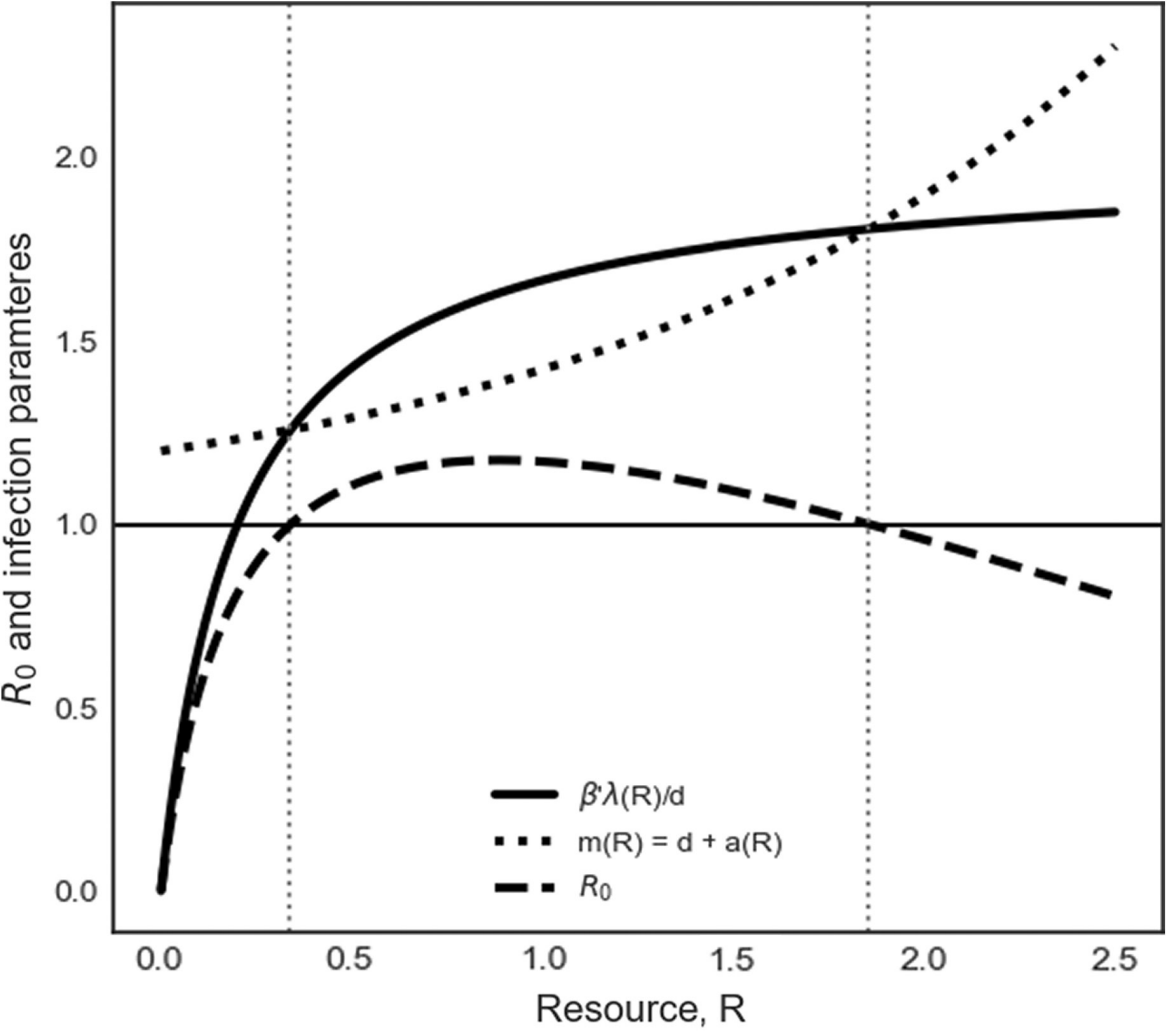
Within hosts, a nonmonotonic *R*_0_ can arise from an interplay of resource impacts on among‐cell pathogen transmission and cell mortality. Within‐host pathogen persistence for the model given in Box 2, Equations 2.4 and 2.5. The solid line is the infected cell production rate when the pathogen is rare; the dotted line is the rate of loss of infected cells due to background cell death and a resource‐dependent immune response; the dashed line shows *R*_0_. Parameters are *d* = 1, *β*′ = 0.01, *λ*(*R*) = 1000/(1 + 5*R*), and *aR*) = 0.2e^0.75*R*^. The region between the vertical dotted lines represents the range of *R* where the criterion for pathogen persistence is met.

Despite the extensive attention the topic of nutrition on infection has received, key gaps remain. Theoretical investigations have generally described resources impacting a single rate or aspect of host or pathogen biology (but see e.g., Hall et al., 2009; Rapti & Cáceres, 2016; van de Waal et al., in review). Yet, the concurrent impact of limited, shared resources on multiple, sometimes countervailing, aspects of host and pathogen growth and interactions (e.g., pathogen transmission, host growth, and host immune defense), suggests the possibility for the emergence of nonmonotonic dynamics along a resource supply gradient (Figure 1). Experimental investigations into the impacts of resource supply on hosts and pathogens have generally focused on a single low and a single elevated resource level. Yet with multiple concurrent effects of resources on both hosts and pathogens, the outcome for host or pathogen fitness (*R*_0_, i.e., the number of new infections expected when an infectious host enters a completely susceptible population) may shift in complex ways across a gradient of resources. For example, the sporulation and pathogenicity of the fungal plant pathogen *Botrytis cinerea* have been documented to change nonmonotonically along a nitrogen supply gradient (Abro et al., 2013). Similarly, in a convincing demonstration using both theory and experiments, nonmonotonic dynamics of a fungal pathogen's fitness were observed along a continuous gradient of resources (algae) available to its *Daphnia* host (Hall et al., 2009).

It remains unclear, however, whether such nonmonotonic dynamics due to concurrent resource dependencies of multiple rates are common across the tree of life. While few experiments have examined both host and pathogen dynamics under multiple rates of resource supply, nonetheless, we motivate this review by describing exemplars of systems with the best available evidence of nonmonotonic relationships between resources and pathogen or host fitness. We complement this empirical perspective by presenting two highly generalized models of within‐ (Box 2) and among‐host (Box 3) dynamics responding to abiotic resources and a model with biotic resources (Box 4) to provide a framework for contextualizing a wide range of empirical results. These models, generated to focus on the range of outcomes arising from concurrent effects of resource supply on multiple biological rates, provide a means to distill the results arising from data‐driven, system‐specific models (Rastetter, 2017; Servedio et al., 2014). We then use these general models to guide a review of the evidence for the dependence of host and pathogen rates (i.e., demographic rates, infection‐related processes) on resources across the tree of life. Our models illustrate that, when combined, these individual resource‐rate relationships may interact to cause nonmonotonic effects on pathogen fitness across a gradient of resource supply. We focus on the role of resources in modifying postinfection pathogen dynamics via host cell chemistry and metabolite production, immune response, size and growth rate, and pathogen transmission. We survey examples from primary producer and consumer hosts spanning terrestrial, freshwater, and marine environments, and we end by pointing to exciting future directions for inquiry.

## 2 EXAMPLES OF COUNTERVAILING EFFECTS OF RESOURCE SUPPLY

Countervailing forces inducing nonmonotonic relationships between resource supply and pathogen fitness appear to be at work in producer and consumer taxa across aquatic and terrestrial environments. While empirical studies documenting concurrent resource effects within host individuals and populations are scarce, the diversity of environments and host and pathogen taxa within existing examples suggests the generality of these interactive effects. We begin by presenting a few examples where these dynamics appear to be at work.

### 2.1 Fungal pathogen dynamics in an aquatic invertebrate host

In a study of *Daphnia*, algal (resource) quality, and fungal pathogens (*Metschnikowia bicuspidate*), the birth rate of uninfected hosts and spore production increased linearly with resource quality while host susceptibility to infection by spores declined linearly (Hall et al., 2009). Higher birth rate and spore production increased *R*_0_ while lower susceptibility decreased *R*_0_. These opposing processes led to a concave relationship between *R*_0_ and resource quality that reflected epidemic patterns observed in midwestern lakes (Hall et al., 2009). Thus, nonlinear relationships between *R*_0_ and resource availability can arise from opposing monotonic relationships between aquatic resources and components of *R*_0_.

### 2.2 Fungal pathogen dynamics within a terrestrial plant host

Opposing resource relationships may help to explain variable effects of nitrogen (N) supply on individual plant susceptibility to the necrotrophic fungal pathogen *Botrytis cinerea* (Lecompte et al., 2010). While various studies have captured inconsistent effects of N supply on *B. cinerea* infection across host species and pathogen strains (Hoffland et al., 1999; Lacrampe et al., 2020; Verhoeff, 1968), nonmonotonic relationships between N supply and both sporulation and pathogenicity were observed for single host‐single pathogen pairings (Abro et al., 2013). In this system, N supply has been linked to sugar‐dependent plant defenses and expression of *B. cinerea* virulence genes (Lacrampe et al., 2020), opposing processes that may help explain the nonmonotonic relationships.

### 2.3 Viral pathogen dynamics in a terrestrial vertebrate host population

A model parameterized with data from feral cats and feline leukemia virus demonstrated that isolated monotonic, saturating relationships between fecundity, mortality, contact rate, or transmissibility and resource availability could lead to monotonic and saturating relationships between *R*_0_ and resource availability (Becker & Hall, 2014). When these nonlinear (and sometimes opposing) resource relationships were combined, the emergent relationship between *R*_0_ and resource availability took on a range of nonlinear forms, including saturating, concave, and convex, depending on how strongly resource availability reduced transmissibility and virulence (Becker & Hall, 2014).

## 3 EFFECTS OF RESOURCE SUPPLY ACROSS THE TREE OF LIFE

**FIGURE 2:**
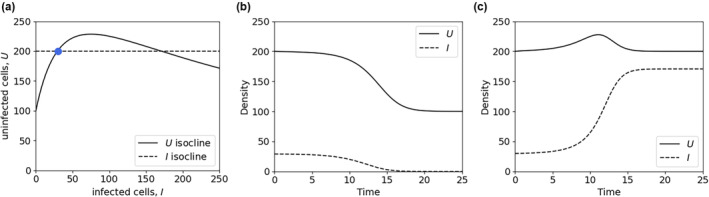
Within hosts, multiple equilibria and alternative states can occur across a continuous gradient of resources. (a) Isoclines of the model in Box 2 (Equations 2.4 and 2.5) with *d* = 1, *m* = 2, *β*' = 0.01, and $\lambda =\left(100+8I\right)/\left(1+0.01I\right)$ produce multiple equilibria. Here, resources are implicit, in that host production of healthy cells (*U*) may require greater resource inputs to support higher cell division rates (see Box 2). The blue dot, a locally unstable equilibrium, indicates the location of simulations shown in panels (b) and (c) when the equations are solved, starting *U* at its equilibrium. The other equilibrium is locally stable. (b) When *I* is initially 29, just below its lower equilibrium (blue dot, panel a), the pathogen cannot persist (the no‐infection equilibrium). (c) When *I* is initially 30, just above its lower equilibrium, the pathogen persists within the host.

**FIGURE 3:**
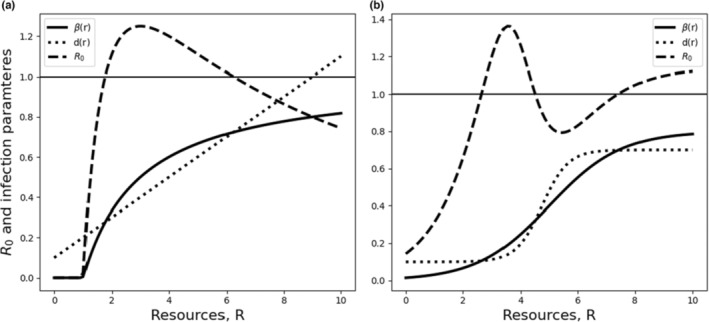
Among hosts, a nonmonotonic *R*_0_ can arise when resource supply increases pathogen transmission and host mortality. SI model (see Box 3) with constant total population (*K* = *S* + *I*) and abiotic resource (nutrients, *N*), so $dI/dt=\left(\mathrm{\beta S}-d\right)I=\left[\beta \left(K-I\right)-d\right]I$, where β is the transmission rate and *d* is the removal rate (mortality and, if applicable, recovery). When *R*_0_ is less than 1 (thin solid horizontal line), the pathogen cannot persist. (a) *K* = 1, $\beta =\max\left\{0,0.5\left(R-1\right)/\left[1+0.5\left(R-1\right)\right]\right\}$ (solid curve) and *d* = 0.1 + 0.*R* (dotted line). Also shown is $R_{0}=\mathrm{\beta K}/d$ (dashed line). If *N* is in the region where *R*_0_ > 1 (here, approximately 1.8 to 6.1), the pathogen can increase when rare, and *I* grows logistically to $K\left(R_{0}-1\right)/R_{0}$, which is its stable equilibrium; otherwise, the pathogen cannot increase when rare and declines to 0. (b) Same as (a) except $\beta =0.8/\left(1+\exp\left[-0.8\left(R-5\right)\right]\right)$ and $d=0.6/\left(1+\exp\left[-2.2\left(R-5\right)\right]\right)$.

**FIGURE 4:**
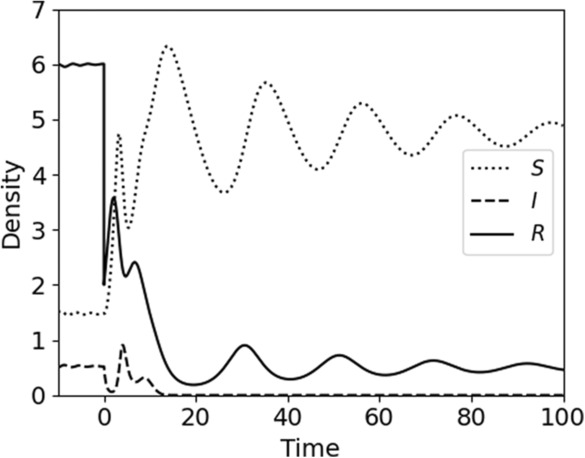
Alternative stable states can arise when transmission depends on resource quantity. In a model (see Box 4) with a biological resource (*R*), a host that consumes that resource (*S*) and can become infected by a pathogen (*I*), and predation (or other density‐independent mortality), an external shock can send the system into an alternative stable state. Here, we illustrate the system initiated at equilibrium with the pathogen present, where *R* = 6. At *t* = 0, an external shock reduces *R* to 2. Susceptible hosts increase and, concurrently, reduced transmission causes a decline in infected hosts. Infected and susceptible hosts cycle briefly, before infection is eliminated. Here, *r* = 1, *K* = 10, *a* = 0.2, *b* = 1, *m* = 0.1, *α* = 3, *β* = *γR*, *γ* = 0.35.

We proceed by examining a wide range of examples of resource impacts on key rates, both within‐host individuals (Box 2) and across a host population (Box 3). The models we present here provide a highly generalized synthesis of a range of existing models (e.g., Becker et al., 2015; Becker & Hall, 2014; Cressler et al., 2014; Hite & Cressler, 2019), illustrating that when resources impact multiple aspects of host and pathogen biology, this relationship may induce nonmonotonic dynamics within hosts (Box 2, Figures 1 and 2) and in the population dynamics of hosts and their pathogens (Box 3, Figure 3). These generalized models clarify that when resources simultaneously impact multiple countervailing rates (e.g., pathogen transmission and host mortality), nonmonotonic, nonintuitive results can arise. For example, both very low and very high resources may facilitate host population growth by suppressing pathogen growth both within‐host individuals (Box 2) and in host populations (Box 3), whereas intermediate resources may favor pathogen proliferation within hosts and host populations (Figures 1 and 3, Boxes 2 and 3). Multimodal responses also can emerge, in which pathogens persist at quite low and high resource levels, but not in zones in between. Our generalized modeling approach builds from previous work on trophic interactions (Packer et al., 2003) to show that resource‐mediated effects also can lead to surprising effects of predation and other causes of mortality on disease prevalence (Box 4), including the possibility of alternative states (Figures 2 and 4).

Our distilled models illustrate that the invasion or persistence of a pathogen within‐ and among hosts depends on ratios of pathogen transmission to host and pathogen vital rates as a function of resources. The outcome may at times be unimodal, such that increasing nutrients may lead to an initial increase and subsequent decline in the epidemic potential or infection prevalence of a pathogen over a resource gradient. For example, within hosts, if an increase in the supply of a resource concurrently increases the production rate of new cells (*λ*) but induces an even more rapid increase in the infection‐induced cell death rate (*a*), the population of infected host cells may follow a nonmonotonic trajectory, with *R*_0_ meeting the criterion for pathogen persistence only at intermediate resource concentrations (Figure 1, Box 2). Thus, even where nutrients have monotonic effects on individual rates, the combined effects of the same nutrient on multiple rates in the host‐pathogen system may lead to nonmonotonic outcomes along a continuous resource gradient on the persistence of infected cells within hosts (Figure 1) or the persistence of infected hosts across host populations (Figure 3).

### 3.1 Effects of resource supply on within‐host dynamics

Decades of empirical studies demonstrate that variation in resource supply rates to hosts across the tree of life can influence postinfection pathogen dynamics within a host. For example, early work on within‐host pathogen dynamics in guinea pigs established that without additional dietary iron, two different bacterial species, *Clostridium welchii* (Bullen et al., 1967) and *Escherichia coli* (Bullen et al., 1968), declined to extinction, whereas with iron supplementation the populations of both pathogens increased rapidly after inoculation. This role of nutrient metals such as iron, zinc, and copper in limiting bacterial infection has been demonstrated to be quite general across animal hosts and their bacterial pathogens (Bullen, 1981; Li et al., 2019; Murdoch & Skaar, 2022). Similar within‐host dynamics in response to host nutrient supplementation have been documented for fungi infecting hosts ranging from plants (Marschner, 2011; Walters & Bingham, 2007) to arthropods (Bedhomme et al., 2004) and mammals (Li et al., 2019). Viral production rate also is limited by host cellular nutrient content in hosts across the tree of life, from bacteria (e.g., T4 phage in *E. coli*, Hadas et al., 1997), ciliates (Clasen & Elser, 2007), and phytoplankton (Cheng et al., 2015; Maranger & Bird, 1995; Wilson et al., 1996) to plants (Kaplan & Bergman, 1985) and vertebrate animals (e.g., SHIV, Smith et al., 2015). In each of these cases, host nutrient supply increasingly favors pathogen production (*k*, Box 2) over loss (*c*), likely altering both the epidemic potential (*R*_0_) and total number of infected cells at equilibrium.

BOX 2 **Within‐host infection dynamics.**

Resources can influence within‐host pathogen dynamics in two distinct ways. First, pathogens may deplete resources, possibly competing with the host (Smith & Holt, 1996) via, for example, competition with immune cells (Cressler et al., 2014; Greenspoon et al., 2018). In this case, within‐host resource levels are explicitly tracked in dynamical models. Alternatively, resources to the host could modulate proliferation and loss of pathogens or infected cells. In this case, resources may be regulated by whole‐organism homeostatic mechanisms. For example, sugar in a host's bloodstream can be physiologically controlled, and that energy may determine the host's ability to replenish healthy cells or mount defenses against infection.

Here, we create a generalized model of resource‐dependent within‐host processes by modifying a classic within‐host model for viral dynamics (Nowak & May, 2000), as follows:

(2.1) $$\frac{dU}{dt}=\lambda \left(R\right)-d\left(R\right)U-\beta \left(R\right)\mathrm{UV}$$

(2.2) $$\frac{dI}{dt}=\beta \left(R\right)\mathrm{UV}-m\left(R\right)I$$

(2.3) $$\frac{dV}{dt}=k\left(R\right)I-c\left(R\right)V$$

where *U* is the density of uninfected cells, *I* is the density of infected cells, and *V* is the density of free pathogen. Uninfected cells recruit at rate *λ* (per unit time; all other rates are per capita), die at rate *d* and are infected at rate *βV*, producing infected cells. Infected cells produce pathogen at rate *k* while living, and they die at rate *m*. Here, *m(R)* = *d(R) + a(R)*, where *a(R)* is additional cell death due to infection, which can depend upon resource levels (e.g., host defensive responses clearing infected cells, or higher mortality of infected cells). The free pathogen dies at rate *c* (clearance rate). Here, parentheses around *R* indicate that a parameter can vary with resource supply.

By assuming the free pathogen equilibrates rapidly compared to changes in host cell numbers, we can set d*V/*d*t* = 0 to reduce the model (and for notational convenience, making resource dependencies implicit, rather than explicit) to

(2.4) $$\frac{dU}{dt}=\lambda -dU-\beta^{\prime }\mathrm{UI}$$

(2.5) $$\frac{dI}{dt}=\left(\beta^{\prime }U-m\right)I$$

where $\beta^{\prime }=\frac{\mathrm{\beta k}}{c}$ is an effective transmission rate. With no infection, the equilibrium density of uninfected cells is $U^{*}=\frac{\lambda }{d}.$

The pathogen population can increase when rare if and only if $\frac{\beta^{\prime }\lambda }{d}>m$. If this holds, then the system has an equilibrium consisting of both uninfected and infected cells. This condition for increase of the pathogen when rare, spreading among cells of an individual host, is comparable to that depicted in Box 3 (spread among‐host individuals). Thus, resource supply can generate comparable nonmonotonic patterns at both scales.

Within‐host infection dynamics depend on the input rate of susceptible, healthy cells, *λ*, which may interact with resources to generate nonmonotonic outcomes. For example, increasing *R* supply can increase susceptible cell production, facilitating pathogen establishment. However, increased resources could concurrently allow the host to mount a more robust defense. Figure 1 (main text) provides a hypothetical example. If host cell production saturates with increasing resources, whereas host defense accelerates, then a pathogen may persist only at intermediate resource availability. Likewise, if the nutrient content of healthy cells increases with resource supply, this enriches the growth environment for pathogens, boosting *β*'. If host defenses deplete free‐living pathogens, this reduces *β*'.

Figure 2 (main text) illustrates a hypothetical example of an emergent nonmonotonic relationship with alternative states. At low resource supply, *R* constraints on λ or *β* (or both) cause healthy cell production to be too low, and the pathogen cannot invade (Figure 2b). With increased resource, *R* effects on *λ* or *β* create another equilibrium where the pathogen population is sustained (Figure 2c). Additional complications may arise if host defenses (embedded in clearance rate, *c*) depend upon *R*. If increased resources boost defense, the I‐isocline in Figure 2a increases (i.e., the pathogen requires more uninfected cells to invade), reducing the equilibrial level of within‐host infection or even eliminating the pathogen entirely.

Hosts and pathogens often compete for shared resources, so empirically separating the direct effects of nutrients on pathogen dynamics from the indirect effects via nutrient‐induced changes to host physiology or defense raises significant challenges. If hosts were nonbiological habitats for pathogens, such as a chemostat or petri dish, increased resource supply would be expected to enhance pathogen growth and replication. Some pathogens can be grown in media, allowing isolation of some of the direct effects of resources on pathogen population size. For example, Neri et al. (2011) manipulated nutrient concentrations in agar media, creating lattices of resource heterogeneity, to quantify host‐independent growth of the fungal plant pathogen *Rhizoctonia solani*. In that case, increased nutrient supply tended to slow fungal spread (Neri et al., 2011). However, many pathogens cannot grow or replicate in media, requiring alternative approaches to determine the direct effect of elemental nutrition on pathogen dynamics. *Vibrio cholerae*, the causative agent of cholera, and many other bacterial pathogens (Bullen, 1981) demonstrate their reliance on iron for growth through investment in iron acquisition systems that overcome host sequestration of iron (Parrow et al., 2013; Rivera‐Chávez & Mekalanos, 2019). It is becoming clear that investment in host mechanisms to defensively sequester – and concurrent pathogen mechanisms to access – growth‐limiting nutrients is a general phenomenon for hosts and pathogens across the tree of life (Gerwien et al., 2018; Murdoch & Skaar, 2022; Pike et al., 2019; Weiss & Carver, 2018). In fact, this competition that can sway the interaction to favor outcomes for the host or the pathogen is so broadly relevant that its study is not restricted to the field of ecology. The study of “nutritional immunity” in human medicine has arisen to develop mechanistic understanding to successfully manipulate competition for dietary metals between human hosts and their pathogens (Murdoch & Skaar, 2022). This investment in resource acquisition by both hosts and pathogens points to the importance of element supply to hosts for pathogen growth. However, defense investment by hosts remains a key component to understanding the net role of nutrient supply in modulating host‐pathogen interactions.

#### 3.1.1 Individual host defense and host–microbe interactions

Resource supply to hosts can induce a wide range of effects on host defense and host–microbe interactions (Alexander & Turnbaugh, 2020; Pike et al., 2019; Smith & Holt, 1996). With increasing resource supply, many hosts increase the production of defense‐related enzymes, proteins, and gene expression, improving immune defense in hosts spanning animals (Becker et al., 2015; Cypher & Frost, 1999; Tourkochristou et al., 2021) and plants (Borer et al., 2022; Veresoglou et al., 2013). This is consistent with immune responses directly suppressing pathogens (e.g., increased *c* with higher *R*, Box 2) or reducing the competitive ability of pathogens for resources shared with host cells via impaired resource‐use efficiency (suppression of *k* with higher *R*, Box 2; and see van de Waal et al., in review). However, elevated resources also have been observed to reduce immune function and suppress defense investment in plant and animal hosts (Berg & Koskella, 2018; Cornet & Sorci, 2010; Hirschberger et al., 2021; Pike et al., 2019). For example, nitrogen and phosphorus can weaken the cell walls of a wide range of autotrophs, a first line of defense against pathogens (Borer et al., 2022). In animal hosts, diets high in carbohydrate or fat (~high C) or low in protein (~low N or P) can impair immune function and survival (Becker et al., 2015; Hirschberger et al., 2021; Miller & Cotter, 2018). While dietary intake of, for example, carbohydrates may be manipulated by hosts, pathogens of animal hosts also can manipulate host metabolism to reduce blood glucose (C), a critical resource for a host's immune upregulation (Freyberg & Harvill, 2017). Starvation and anorexia in both vertebrate and invertebrate hosts also can reduce nutrient supply, weakening virulence and providing defense against pathogens (Ayres & Schneider, 2009; Hite & Cressler, 2019; Murray & Murray, 1979), further underscoring the importance of host‐pathogen competition for resources in controlling infection and disease.

More complex within‐host interactions may also underpin some of these observed relationships. Empirical work in a tomato host has demonstrated that a pathogen's identity and traits can determine the impacts of the supply rate of a nutrient (Hoffland et al., 2000), an observation consistent with defenses varying with the relative competitive abilities of hosts and microbes for shared resources (Cressler et al., 2014). The host's microbiome, which can serve as an important line of defense against infection, can shift in composition with low or high resource supply rates, creating opportunities for pathogens to invade (Lopez & Skaar, 2018). Host‐pathogen resource competition via signal manipulation with a host's microbiome also is gaining increasing attention, particularly in mammalian hosts (Cameron & Sperandio, 2015; Murdoch & Skaar, 2022, see Section 4, below). While our generalized model (Box 2) does not describe these intracellular details, it does highlight the key role of resource supply on both resource competition (effect of *R* on *k*, Box 2) and immune investment (effect of *R* on *c*, Box 2), both of which influence epidemic potential (*R*_0_). However, investment of growth‐limiting resources into defense can be costly for hosts, potentially inducing trade‐offs between growth and defense across a gradient of nutrient supply.

#### 3.1.2 Growth & defense trade‐offs in host individuals

As reviewed by Borer et al. (2022) for primary producers and Becker et al. (2015) for consumers, resources can enhance both host growth (increased *λ*, Box 2) and defense (decreased *β* or *k*, increased *c* or *m*, Box 2), therefore, modifying the outcome of infection within host individuals. Host growth rate may directly limit pathogen growth rate because host cellular metabolic rate is a key constraint for the within‐host replication of many pathogens (Freyberg & Harvill, 2017; Nørgaard et al., 2021; Selman & Yahampath, 1973; Smith et al., 2015; Weinbauer, 2004), so factors, such as resources, that increase host growth can also facilitate pathogen growth. Resource‐enhanced growth of plants can create more space for pathogen colonization and replication (Borer et al., 2022), a response observed with viruses in grasses (Whitaker et al., 2015). However, sufficient nitrogen can concurrently allow a plant to increase the production of defense‐related enzymes, proteins, and gene expression (Borer et al., 2022), suppressing pathogen growth while also promoting host growth. Similarly, energy, protein, and nutrients can control both growth and immune functioning in bacterial (Weinbauer, 2004) and animal hosts (Becker et al., 2015; Hirschberger et al., 2021; Nørgaard et al., 2021). For example, increased immune investment with resource availability in vampire bats can support an increased host population while suppressing the pathogen via enhanced immune responses (Becker et al., 2018). The simultaneous operation of these two effects opens the ground for a wide range of dynamical outcomes, as explored in the Boxes.

In both plants and animals, the tension between investment of growth‐limiting resources into growth or defense can lead to trade‐offs that likely underlie these seemingly opposite effects of nutrient supply on pathogen dynamics. In birds, allocation of energetic resources to feather growth trades off with immune response (Ben‐Hamo et al., 2017), and humans experience a similar energetic tradeoff between immunity and childhood growth (Urlacher et al., 2018). In herbaceous plants, the effect of nitrogen can have opposite effects on fungal infection severity, depending on the host and pathogen species (Veresoglou et al., 2013), likely as a function of heterogeneity across taxa in this allocation tradeoff. Across the tree of life, larger autotrophs tend to allocate fewer resources to chemical defenses (Borer et al., 2022) and the time animals spend foraging for food often comes at the expense of time spent on behavioral defenses, such as grooming (Becker et al., 2015). Thus, trade‐offs between resource investment into growth or defense provide a theater in which the simultaneous, but countervailing, effects of nutrient supply on hosts and pathogens can play out. In each of these cases, nutrient supply to hosts increasingly favors host growth (increased *λ*, Box 2) while also allowing investment in defense (reduced *β* or *k*, increased *c*). Because the within‐host pathogen population relies, in part, on the relationship between these host attributes and pathogen replication and clearance rates, the relative influence of nutrients on each of these rates will determine infection dynamics across a gradient in nutrient supply, with a wide range of parameter space in which the outcome is expected to be nonmonotonic, possibly even generating alternative stable states (Boxes 2 and 3, Figure 2).

### 3.2 Effects of resource supply on among‐host dynamics

Our generalized model of susceptible and infected hosts demonstrates that where nutrient supply impacts concurrent rates differently, nonmonotonic relationships also can emerge at the scale of host populations (Figure 3, Box 3). This is analogous to the within‐host model (Box 2), and these relationships often arise from nutrient effects on individual host physiology (Section 3.1), although other factors also can be important, such as how resource supply affects contact patterns among individuals via plastic behavioral responses and “bottom‐up” resource effects on host abundance. A large body of theoretical and empirical work has examined the impact of nutrient supply on pathogen dynamics at the scale of host populations across a wide range of systems (Becker et al., 2015; Becker & Hall, 2014; Borer et al., 2022). We do not attempt to review this entire literature. Rather, we focus on examples of the resource dependence of vital rates that, acting together, could lead to nonmonotonic changes in the prevalence or basic reproductive number (*R*_0_) of pathogens in host populations across kingdoms and habitats.

BOX 3 **Among‐host transmission of a nonregulatory pathogen.**

To illustrate some ways that resources can influence infection dynamics, we assume that a host population is regulated to a carrying capacity, *K*, by factors other than infectious disease. For instance, consider space‐limited organisms (canopy trees in a tropical forest, or barnacles crowding a rocky shore), where individuals produce vastly more offspring than can be accommodated when a habitat is saturated. Infectious disease could affect relative fitness (e.g., death rates) without causing an impact on realized equilibrial population size. An alternative scenario with the same effect on the host population might be agriculture, where an external agent determines host numbers (e.g., size of a cattle herd, number of corn plants in a field). Thus, in the scenarios we examine here, individual hosts are either susceptible, or infected, with respective densities *S* and *I*. Because of our assumption about host population regulation, *S + I = K*. In other words, when individuals die, for instance because of virulent infection, they are immediately replaced by susceptible recruits into the population. This assumption permits a simplification of the math, helping to illuminate the qualitative impacts of resources on system dynamics

Infection dynamics are governed by transmission from infected to healthy hosts, and by loss of infection due either to host death or recovery. For simplicity, we assume that hosts become infected via density‐dependent transmission, *βSI*. If individuals recover, they re‐enter the susceptible class. Death occurs for healthy hosts at rate *d*. Infection increases this death rate by an increment *a*, and some infected individuals may recover to become susceptible again, at rate *e*. The total per capita loss rate of infection (per infected individuals) is *m = d + a + e*. The dynamics of the infection are thus described by

(3.1) $$\frac{dI}{dt}=\mathrm{\beta SI}-mI=\beta \left(K-I\right)I-\mathrm{mI}$$

This logistic growth equation describes the infected class of the population. The intrinsic growth rate of the equation (evaluated when the infection is rare, so *I* is approximately zero) is r=βK−m. The reproduction rate of the infection is $R_{0}=\frac{\mathrm{\beta K}}{m}$. The equilibrial density of the infection is $I^{*}=K-\frac{m}{\beta \;}$, which means equilibrial prevalence is $\frac{I^{*}}{K}=1-\frac{1}{R_{0}}$.

As in Box 2 (within‐host) model, any of these parameters could depend upon resource supply; here, we denote resource abundance by *R*, and replace each parameter with a function of *R*. How the various metrics describing the infection (e.g., reproduction ratio, equilibrial infected abundance or prevalence) vary with resource level depends upon their functional relationships. The pathogen persists if

(3.2) $$\beta \left(R\right)K\left(R\right)>m\left(R\right)+e\left(R\right)$$

Consider first a host regulated by a factor other than this focal resource. Let us imagine that increased resources lead to greater within‐host pathogen loads. This could increase transmission, but it also may increase mortality. The functional relationship between these two parameters could differ, either quantitatively or qualitatively.

Figure 3 (main text) shows an example, where *β* and *d* both increase with *R*. In the first case (Figure 3a), mortality increases linearly with *R*. Transmission, however, requires a minimal amount of *R*, but with increasing resource levels, transmission increases, then saturates. A nonlinearity in transmission such as this might arise via infection‐induced changes in host behavior. If transmission requires contact, and infected individuals move more sluggishly, fewer contacts may occur in a high resource, infected population. The reduced contact despite elevated nutrients can partially compensate for the increased probability of infection, per contact. Similar changes in transmission dynamics may occur with resources supplied to plants, if the resources, for example, affect vector abundance or behavior. Vector numbers may rise with resources to plants (increasing e.g., plant quality), but be limited by extrinsic factors (e.g., mortality from parasitoids). At low nutrient levels, the reproductive ratio for the pathogen (*R*_0_) is less than one (Figure 3), so the pathogen fades away. At very high levels, the ratio is again less than one, because death of infected hosts outstrips transmission. In this example, along an environmental gradient in nutrient availability, the pathogen would persist at intermediate nutrient levels. Different biological assumptions about the influence of resources on system rates (i.e., different functional forms for components of Equation 3.2), can result in even more complicated nonmonotonic dynamics. For instance, if either transmission or mortality (or both) increase as sigmoidal functions of *R*, a multiphasic *R*_0_ (with e.g., epidemics occurring only at intermediate and high resource supply) can emerge along a continuous gradient of resource (Figure 3b).

#### 3.2.1 Resources and pathogen transmission among hosts

Pathogen transmission (*β*, Boxes 2 and 3) links within‐host to among‐host infection dynamics, encompassing several processes, which may depend on resources consumed by the host. These include the concentration of infective parasite stages produced within infectious hosts, the rate of production of pathogen propagules permitting novel infection, contact between susceptible and infectious hosts or free‐living parasites, and the degree of success of parasite movement among hosts (McCallum et al., 2001). Although transmission is often modeled as independent of resources, empirical examples in both animal (Johnson et al., 2007) and plant (Abro et al., 2013) hosts demonstrate that increasing nutrient supply can substantially alter the rate of pathogen production by infected hosts. For vector‐borne pathogens, increasing environmental resources can release vector populations from growth limitation, increasing population‐level transmission of both plant (Strauss et al., 2020) and animal (Boerlijst et al., 2022; Ostfeld et al., 2006; Pope et al., 2005) pathogens. For directly or environmentally transmitted pathogens, transmission can increase with host population density, a function of population size (next section), and aggregation (e.g., animal behavior or crop spacing; see Section 4, below). If parasites with a free‐living stage can exploit increased available resources within individual hosts, this could increase the reserves in free‐living stages, thus facilitating transmission by increasing persistence time in the environment. We are unaware of any studies that have focused on this issue.

While many examples point to resources supplied to hosts increasing transmission, transmission may be suppressed where resources are invested by hosts in defense (Section 3.1). Because both *R*_0_ and infection prevalence are a function of transmission (*β*), host carrying capacity (*K*), and host mortality (*d*), nonmonotonic dynamics can arise along a resource supply gradient where two or more of these rates vary with resources (Figure 3, Box 3). A positive monotonic and saturating relationship, for example between among‐host transmission rate and resource availability (*β*(*R*), Box 3), and a positive linear relationship, for example between virulence and resource availability (*d*(*R*), Box 3), can lead to a concave relationship between *R*_0_ and resource availability (Figure 3a, Box 3, an example concordant with dynamics shown in the data‐driven model of Becker & Hall, 2014).

#### 3.2.2 Resources, host population size, and pathogen prevalence

Increased environmental resources can increase host resource content, growth, or reproduction, increasing host population size (*K*, Box 3) and, in cases spanning bacteria to vertebrate hosts, also benefitting the infecting pathogen. For example, in a chemostat experiment with *Eschericia coli* and bacteriophage T4, glucose increased the equilibrium concentrations of both the host and the phage (Brendan & Lenski, 1997). A field study showed parallel effects, with higher final bacteria concentration and a higher percentage of virus‐infected bacterial cells when dialysis bags, containing bacteria but permeable to viruses and nutrients, were inserted into a nutrient‐rich river site, compared to a more nutrient‐poor site (Simek et al., 2005; Weinbauer, 2004). In another branch on the tree of life, supplemental feeding of wildlife that increases host population density has been linked to increased bacterial infection prevalence in vertebrates from songbirds (*Mycoplasma gallisepticum*, Moyers et al., 2018) to deer (*Mycobacterium bovis*, Cosgrove et al., 2018; Miller et al., 2003). It is even hypothesized that the rise of agriculture increased human population density and individual interactions, increasing infection prevalence in humans (Rohr et al., 2019; Vlok & Buckley, 2022). Although resource effects on host population size may dominate the dynamics of infection prevalence for some pathogens, if the rate of pathogen transmission (*β*) also varies with resource supply to hosts, nonmonotonic effects of resources on *R*_0_ and infection prevalence in a host population could emerge (Box 3).

#### 3.2.3 Resources and host population demography

The impact of resources on population size (*K*, Box 3) and resulting infection is widespread across the tree of life (e.g., Becker et al., 2015; Borer et al., 2022), with population size arising from the demographic processes underlying population growth rate. Resource supply may have differing impacts on host birth or death rates; however, in most empirical studies, these rates are not (and often cannot be) studied in isolation. Host death, for example, may be influenced by resource availability (susceptible host abundance can be altered by changing mortality rates, thus influencing infection dynamics by shifting transmission). Many population‐level empirical studies focus on extrinsic resource supply, yet steady‐state resource availability to individual hosts (i.e., per capita resource supply) also could indirectly increase if hosts experience elevated mortality rates, reducing population size and thus enhancing the availability of resources to surviving hosts (Smith et al., 2015). Empirical evidence suggests that this pathway can increase host and pathogen population growth rates. In a laboratory experiment with retrovirus SHIV89.6P infection of a human T‐cell line (CEM cells), increasing the dilution rate reduced host cell population size but increased resource availability per cell and the cell population growth rate. The concentration of virions per cell and in the supernatant also increased (Smith et al., 2015). A number of additional experiments with cells and viruses have demonstrated comparable results (Smith et al., 2015), suggesting an intimate link between host mortality, resource availability, and population growth.

Host resource effects also can interact with predation or herbivory, determining infection dynamics. Few empirical studies have combined all these factors, but where resources, hosts, pathogens, and predation were combined in a data‐driven model of an invertebrate host (*Daphnia dentifera*) and its fungal parasite (*Metschnikowia bicuspidata*), a wide range of dynamics were shown to occur, including nonmonotonic outcomes for *R*_0_ arising from the interplay of, for example, the virulence of the pathogen and timing of predation. However, for the parameters used in this model, infection generally increases with resources (Rapti & Cáceres, 2016). A general model describing a heterotrophic host and its living resource, pathogen, and predator builds from this system‐specific model to clarify that even when predators act as forces of density‐independent mortality and the resource species impacts only a single parameter (e.g., *β*(*R*), Box 4), nonmonotonic dynamics and alternative states can emerge that depend on resource density (Figure 4). Together, these models (within‐host, among‐host, and in a system with a predator or other extrinsic cause of mortality) suggest that, as in many host‐pathogen systems, when rates depend on resources, a wide range of unexpected, nonmonotonic outcomes can emerge.

BOX 4 **Dynamic resource dependencies and predation impacts on infectious disease dynamics.**

The interplay of resources and predation (a.k.a. bottom‐up and top‐down forces) has been a longstanding focus in community ecology. Packer et al. (2003) combined simple models with illustrative empirical examples to argue that predators, by eliminating infected hosts, could lower the basic reproductive number and equilibrial prevalence of infectious diseases. However, this conclusion ignored cascading effects of predation upon basal resource availability. Other authors have noted the importance of the interplay of infection, predation, and resource competition for specific pathogens (e.g., schistosomiasis, Civitello et al., 2018). Here, we illustrate some of the possible consequences of including a dynamic resource, using the general model:

(4.1) $$\frac{dS}{dt}=\text{baSR}-\mathrm{\beta IS}-\mathrm{mS},\beta \equiv \beta \left(R\right)$$

(4.2) $$\frac{dI}{dt}=\mathrm{\beta SI}-\left(m+\alpha \right)I$$

(4.3) $$\frac{dR}{dt}=\mathrm{rR}\left(1-\frac{R}{K}\right)-a\left(S+I\right)R$$

Here, *S* and *I* represent susceptible and infected hosts. Generation of susceptible hosts depends on resource consumption (*aSR*), which is scaled by the conversion rate *b*. *R* represents a dynamic, biological resource (e.g., grass, phytoplankton) that grows logistically. For simplicity, the consumer has a linear functional and numerical response to this resource. Predation acts as a density‐independent mortality term, *m* (the model could also generalize to other stressors, which influence mortality rates). Infection increases the death rate of hosts (*α*) and prevents births. Here, transmission (*β*) is a function of resource availability, reflecting, for example, a change in the internal resource state of individual hosts or how contact rates shift with changes in resource abundance.

In the absence of infection, the system settles to an equilibrium given by

(4.4) $$R^{*}=\frac{m}{\mathrm{ab}},S^{*}=\frac{r}{a}\left(1-\frac{m}{\mathrm{abK}}\right)$$

The rate of increase of the pathogen, when it is rare and the host is at its uninfected equilibrium, is

(4.5) $$r_{\text{pathogen}}=\beta \left(R^{*}\right)\frac{r}{a}\left[1-\frac{m}{\mathrm{abK}}\right]-\left(m+\alpha \right)$$

An increase in predation (increased *m*) reduces susceptible host numbers, thus increasing resource availability. The consequences of predation (reduced *S* and increased *R*) can alter the rate of disease transmission. The condition for an increase in mortality to increase *r*_pathogen_ is

(4.6) $$\frac{d\beta }{dR}>\frac{\beta \left(R\right)r+a^{2}\mathrm{bK}}{r\left(K-\frac{m}{\mathrm{ab}}\right)}$$

This can hold if at low *m* (low predation cascades to low ambient resource levels), transmission is low, and the resource growth rate *r*, is high. This inequality becomes is less likely when *m* is large, particularly if the impact of resources on the transmission rate (the left term in the inequality) saturates. This model demonstrates that increased predation can indirectly boost the initial spread of an infectious disease. These results reveal the generality of conclusions reached in a general model with implicit resources (Smith & Holt, 1996) and in an individual‐based model for schistosomiasis (Civitello et al., 2018).

Because *R* has counteracting effects on the host through host growth and pathogen transmission (and *R* is a dependent variable of the system), alternative stable states can arise. Figure 4 (main text) shows an example in which a pathogen initially regulates a host to low numbers, leading to abundance of the resource. This high *R* supports high transmission and effective regulation of the host by the pathogen. However, if a disturbance reduces resource abundance, disease transmission is also reduced. In this case, host numbers rise, reducing *R* and retaining it at a new, low level. Because transmission is resource‐dependent, the pathogen is driven locally extinct.

## 4 TAKING STOCK: SUMMARY AND OPPORTUNITIES FOR FUTURE WORK

Our generalized, system‐independent models (Boxes 2, 3, 4) demonstrate that nonmonotonic outcomes for infection can arise from interactions of resources with two or more demographic processes. Individually, each process can have a simple relationship with resources (e.g., linear), but even two simple, countervailing effects can generate nonlinear dynamics for pathogen persistence, switches in stability, and alternative stable states. Paired with examples of such host and pathogen resource relationships from across the tree of life, this suggests that the joint effect of concurrent, resource‐dependent rates giving rise to nonmonotonic infection dynamics is likely to be common in natural systems.

Our literature survey also points to several opportunities for future work. Both biotic and abiotic factors may shape the resource dependence of rates, and a variety of empirical and theoretical gaps remain to be filled to better understand the role such dependencies play in infection dynamics. Our synthesis suggests that these gaps remain because, on one hand, theoretical investigation into resource effects on individual rates has been well‐explored, but less attention has been given to emergent dynamics along resource gradients when multiple rates are concurrently impacted. On the other hand, empirical investigations have generally focused on responses at single resource supply points (e.g., comparing “low” to “high” resources), rather than at multiple points along a gradient. Here, we summarize some areas in which additional realism could potentially provide further opportunities for examining the prevalence and implications of countervailing effects of resources on key infection rates.

*Nutrient ratios* have been empirically demonstrated to control host immune function and the dynamics of pathogens across the tree of life (Clasen & Elser, 2007; Maat et al., 2014; Maat & Brussaard, 2016; Ponton et al., 2020; Wilson et al., 1996), and stoichiometrically explicit models can predict different dynamics compared to those predicted by single resource models, such as those presented here. Resource ratio theory (Tilman, 1982) applied to pathogens (Smith & Holt, 1996) provides a strong framework for interpreting the invasion and persistence of many pathogens of vertebrate hosts. A Droop modeling approach is frequently applied to describe the dependence of phytoplankton growth on ratios of elements (Droop, 1968) and the Droop model has been employed in modeling infection dynamics where hosts and pathogens share resources (Borer et al., 2022; Pell et al., 2019; van de Waal et al., in review). Expanding the current models to include dependence of key rates on elemental ratios would provide the opportunity to address pressing global change questions, such as how rising atmospheric CO_2_ may affect the dynamics of plant virus replication along gradients of N‐ and P‐fertilization.

*Nutrient interactions with temperature* via, for example, host metabolic changes may alter the dynamics of infection. Although temperature can alter the balance of host defense and growth investment (Padfield et al., 2020), infection has to our knowledge not been paired with temperature along a gradient of nutrients in a single study. However, studies of a single host species provide an example of their potential combined effects. In a ciliate host, *Paramecium bursaria*, with an endosymbiotic green alga (*Chlorella*), temperature alters the host‐endosymbiont relationship. At the same time, nutrient supply limits *Chlorella* and *Paramecium* growth (Kodama & Fujishima, 2012) as well as viral infection of *Chlorella* (Clasen & Elser, 2007). While this is a single example, it suggests that gradients of temperature and nutrients may have countervailing impacts on multiple rates of controlling infection.

*Changing resource effects with infection timing* may play out in a variety of ways to alter resource impacts on infection dynamics. Host metabolism changes with age and size, and pathogen dynamics vary with inoculation timing as a function of host age, physiological state, and nutrient status in a wide range of animal (Tate & Graham, 2015) and plant (Bachand & Castello, 1998; Borer et al., 2022; Yamauchi et al., 2017) hosts. If both healthy cell recruitment (*λ*) and infected cell death (*m*) increase with nutrients, but healthy cell recruitment is greatest in young hosts, slowing with age, then even our simple model suggests that pathogens could most easily invade young hosts (*R*_0_ criteria) and the prevalence of infected cells (I*) could change nonmonotonically with age. In addition to the absolute quantity of resource, the timing of resource supply across life cycles could have substantial impacts on the processes we have identified here.

*Behavior* may interact in a variety of ways with the nutrient environment to change host density and infection dynamics. Supplemental feeding frequently increases host aggregation, enhancing disease transmission, especially of density‐dependent pathogens, by increasing per capita contacts between susceptible and infected hosts (*β*) and altering the local densities of susceptible hosts (*K*) (Becker et al., 2015; Civitello et al., 2018). However, some cases of supplemental feeding suppress disease transmission by directing wildlife away from specific environments or food sources that serve as pathogen reservoirs or by enhancing survival of recovered (and, therefore, immune) hosts (Becker et al., 2015). It is also likely that in some cases, pathogen manipulation of host (Heil, 2016) or vector (Shoemaker et al., 2019) behavior increases transmission as a function of host resources. If increased resource supply to the host facilitates within‐host pathogen population growth, then pathogens might more effectively alter host behaviors to increase resource consumption rates, enhancing their own transmission.

*More complex food webs* with co‐infecting parasites (Smith & Holt, 1996) may generate additional opportunities for the emergence of nonmonotonic dynamics. A growing body of work on microbiome‐immune‐pathogen interactions demonstrates a wide range of resource‐dependent effects of resident microbiota on immunity (Alexander & Turnbaugh, 2020), and the outcome of parasite–parasite interactions also can vary with resource supply (Cameron & Sperandio, 2015; Wale, Sim, Jones, et al., 2017), leading to changes in infection prevalence as a function of resources. For example, limitation of the parasite resource pABA led to competitive suppression of a drug‐resistant malaria strain by a drug‐susceptible malaria strain in mice (Wale, Sim, Jones, et al., 2017). In a related experiment, the drug‐susceptible malaria strain reduced within‐host density of the drug‐resistant strain most at the lowest and highest pABA supply rates out of four levels, suggesting a nonmonotonic relationship between resource availability and parasite competition (Wale, Sim, & Read, 2017). Additional insights into the generality of countervailing impacts of resource rates underlying parasite coinfection and competition with a host's resident microbiota would be a valuable contribution to understanding the resource conditions under which pathogen invasion and coinfection are reduced.

*Impacts on virulence evolution*. A large body of theoretical literature examines the proposition that the evolution of virulence is driven by trade‐offs between transmission and mortality impacts on hosts, and by the likelihood of co‐occurrence within individual hosts of competing parasite strains (Cressler et al., 2016). Recent work has demonstrated that resource availability can modulate how this evolutionary process plays out (Lindsay et al., 2023). It goes beyond the scope of this paper to explore the many ways that might happen, but we do note that if reduced resources reduce host population size, one might expect an evolutionary reduction in virulence. As Cressler et al. (2016, p. 925) note, “any mechanism that reduces the density of susceptible hosts will [lead to] a decrease in transmission rate [and] we would expect virulence to decrease as well.” Thus, we suggest that the evolutionary implications of resources for host‐pathogen evolution is another ripe arena for future theoretical and empirical exploration.

*Experimental resource gradients* are a key empirical need raised by most of the examples in this review. Because of the difficulty of creating gradients of many different resource levels, few experiments have employed these. However, our review and models point to the likelihood of nonmonotonic infection responses that will not be captured by a single elevated nutrient treatment, highlighting a key gap to be filled with future empirical work.

## 5 CONCLUSION

Nonmonotonic dynamics that arise because of resource dependence in multiple concurrent – and countervailing – demographic and disease transmission rates have been documented to occur across the tree of life. Empirical evidence demonstrates that rates underlying pathogen prevalence and intrinsic rate of increase (*R*_0_) in hosts from bacteria, phytoplankton, and ciliates to trees and mammals depend on resources. Taken together with the “opportunities for future work” extensions that further increase biological realism, we may expect nonmonotonic effects of environmental nutrients to be the rule, not an exception, in natural systems. This has important implications for the study of infectious disease. First, explicit consideration of concurrent, countervailing effects of nutrients on infection dynamics provides a framework for understanding seemingly opposite effects of nutrients in different conditions or hosts. In addition, observed outcomes or outcomes at a single resource level in empirical studies may not represent the full range of possible outcomes for a host or its pathogen. Second, and related, where resources for hosts are reduced via, for example, effective pollution control, infection may not decline, but may instead proliferate. Understanding the joint effect of resources on the multiple processes underlying infection will provide a framework for predicting these impacts on focal hosts. Finally, to achieve this, resource gradient studies will be critical for uncovering the conditions under which differing outcomes at, for example, intermediate or high resource levels is most likely.

## AUTHOR CONTRIBUTIONS

**Elizabeth T. Borer:** Conceptualization (lead); investigation (equal); methodology (equal); visualization (supporting); writing – original draft (lead); writing – review and editing (equal). **Amy E. Kendig:** Conceptualization (equal); investigation (equal); methodology (equal); visualization (supporting); writing – original draft (supporting); writing – review and editing (equal). **Robert D. Holt:** Conceptualization (equal); investigation (equal); methodology (lead); visualization (lead); writing – original draft (supporting); writing – review and editing (equal).

## Data Availability

No new data were used in this manuscript. All parameter values are listed in figure legends.
